# Investigating Aging Characteristics of Oil-Immersed Power Transformers’ Insulation in Electrical–Thermal–Mechanical Combined Conditions

**DOI:** 10.3390/polym15214239

**Published:** 2023-10-27

**Authors:** Zonghui Yuan, Qian Wang, Zhigang Ren, Fangcheng Lv, Qin Xie, Jianghai Geng, Jianhao Zhu, Fuyun Teng

**Affiliations:** 1 Hebei Provincial Key Laboratory of Power Transmission Equipment Security Defense, North China Electric Power University, Baoding 071003, China; zz261923@163.com (Z.Y.); lfc@ncepu.edu.cn (F.L.); 13582251628@126.com (Q.X.); gengjh@ncepu.edu.cn (J.G.); 17332414296@163.com (J.Z.); 220222213028@necpu.edu.cn (F.T.); 2 Electric Power Research Institute of State Grid Beijing Electric Power Co., Ltd., Beijing 100075, China; wangxy1996@ncepu.edu.cn

**Keywords:** oil–paper insulation, electrical–thermal–mechanical aging, multiphysics coupling, aging characteristics

## Abstract

The condition and health of large oil-immersed power transformers’ insulation have a direct impact on the safety and stability of the power grid. Therefore, it is crucial to investigate the aging characteristics of oil–paper insulation in power transformers. In this study, we developed a computational model for reclosing current calculation and multiphysics coupling models for magnetic-circuit-force, electrostatic field, and temperature field simulations. The calculated aging resulted in a mechanical stress of 8.71 MPa, an electric field strength of 2.26 × 10^6^ V/m, and a temperature of 113.7 °C. We conducted combined electrical–thermal–mechanical aging tests on the oil–paper insulation and measured various insulating paper performance parameters at different aging stages. Our study revealed that both the mechanical and electrical properties of the insulating paper deteriorated in both aging groups. However, the changes were more pronounced in the electrical–thermal–mechanical aging group compared to the electrical–thermal aging group, indicating that mechanical stress accelerated the aging process of the insulating paper. In the early stages of aging, the rate of performance changes in the electrical–thermal aging group was similar to that in the electrical–thermal–mechanical aging group. However, as the aging time increased, the degradation of performance induced by mechanical aging became more significant. This suggests that the insulating paper’s resistance to mechanical damage, specifically short-circuit resistance, noticeably decreased after prolonged aging.

## 1. Introduction

Power transformers are the most critical equipment in power systems, and the insulation condition of their internal oil–paper insulation directly affects the safe operation of transformers [1]. With the continuous development of the power grid, the number of sudden short-circuit faults increases. The short-circuit electromagnetic force can cause winding deformation, thereby causing serious damage to the oil–paper insulation structure.

According to the classification of aging factors, the aging of insulation in transformers can be roughly divided into thermal aging, electrical aging, and mechanical aging [2]. The thermal aging platform constructed in reference [3] studied the thermal aging characteristics of insulating paper in mineral oil and vegetable oil, and the results showed that aging in vegetable oil is slower. The insulating paper thermal aging test platform built in reference [4] studied the degradation law of the degree of polymerization of insulating paper, and the results showed that the rate of degree of polymerization decrease gradually slows down with aging time. Reference [5] estimated the degree of polymerization (DP) and percentage of moisture content (PMCs) in oil-impregnated paper insulation using dielectric response sensing obtained from frequency-domain spectral tests. The insulating paper aging platform constructed in reference [6] studied the microstructure and electrical characteristic changes in insulating paper under different AC/DC composite voltages and found consistency in the degradation mechanism and microstructure of insulating paper under different composite voltages. In reference [7], accelerated thermal aging experiments were carried out on insulating paper at 120 °C and 130 °C, respectively, and the results showed that the crystallinity index and refractive index of cellulose fiber in insulating oil could be used as feasible indexes for the diagnosis of paper degradation. Reference [8] studied the micro-slip wear between the insulation layers of transformers and found that abrupt axial mechanical forces can cause damage to insulating paper, thus affecting its insulation performance. 

The above studies all investigated the effects of single aging conditions on the aging characteristics of insulating paper, while the aging of insulating paper is generally influenced by multiple factors. Reference [9] established an electro-thermal coupled aging platform for aging research of insulating paper and used multivariate regression analysis to analyze the breakdown life of insulating paper, obtaining a lifetime model applicable to electro-thermal aging. Reference [10] also established an electro-thermal coupled aging platform and found that the degree of polymerization decrease in electro-thermal aging samples is faster than that in single-factor thermal aging. Reference [11] studied the frequency-domain dielectric properties of transformer oil–paper insulation under different aging stress conditions and aging times. Reference [12] found that mechanical–thermal coupled aging has a more significant effect on the degradation of insulating paper mechanical properties compared to thermal aging, and the more intense the mechanical vibration, the more significant the decrease in the degree of polymerization and tensile strength of insulating paper. Reference [13] conducted mechanical–electrical–thermal aging tests with experimentally given conditions and obtained an empirical model for the variation in tensile strength of insulating paper under the combined action of heating, electrical, and mechanical forces. 

Most of the aforementioned studies considered the effects of two aging factors in the aging tests, and the experimental conditions were directly given. This paper will consider the combined aging of the electric field, temperature field, and mechanical stress on insulating paper. The values of each parameter will be obtained through simulation and theoretical calculations, which will be more representative of the operating conditions of insulating paper during circuit breaker reclosing.

In this paper, the short-circuit stress, electric field, and temperature field are calculated by setting up the reclosing current calculation model, the magnetic-circuit-force coupling model, and the simulation model of the electrostatic field and temperature field. Based on the above simulation results, the polymerization degree, tensile strength, elongation at break, dielectric loss factor, and breakdown field strength of insulating paper at different aging stages were measured and analyzed.

## 2. Finite Element Simulation of Oil–Paper Insulation

**Figure 1:**
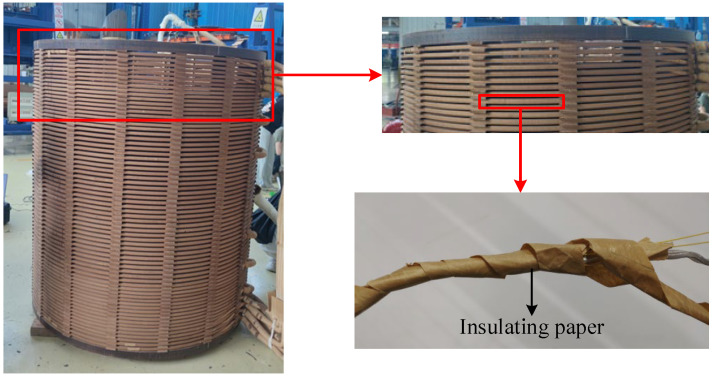
The relationship between insulating paper and winding.

Figure 1 shows the insulating paper in the oil–paper insulation structure. The insulating paper is wrapped on the wire turns, the wire turns are wound to make a winding, and the winding is soaked in the insulating oil. At this time, the insulating paper and the insulating oil form an oil–paper insulation structure.

In this section, the applied forms of field strength, temperature, and mechanical stress are defined by simulation and theoretical calculation. A two-dimensional model of transformer main insulation is established to calculate the general field strength of insulating paper. A Simulink reclosing process simulation model and a 3D magnetic-channel-force coupling finite element simulation model of a transformer are built to calculate the mechanical stress of insulating paper. A two-dimensional model of the transformer temperature field is established to calculate the average winding temperature, and the maximum temperature of insulating paper is finally determined by theoretical calculation.

### 2.1. Electrodynamic Simulation of Oil–Paper Insulation

#### 2.1.1. Reclosing Process Current Calculation

**Figure 2:**
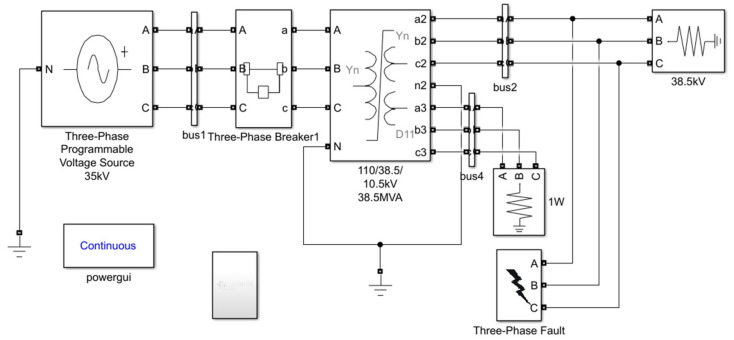
Reclosing Simulink model diagram.

The Simulink reclosing process simulation model built based on SFSZ7-31500/110 transformer is shown in Figure 2. The rated voltage is 110/38.5/10.5 kV, and the parameters of load loss P_k_, no-load loss P_0_, short-circuit voltage percentage U_k_%, and no-load current percentage I_0_% are shown in Table 1.

**Figure 3:**
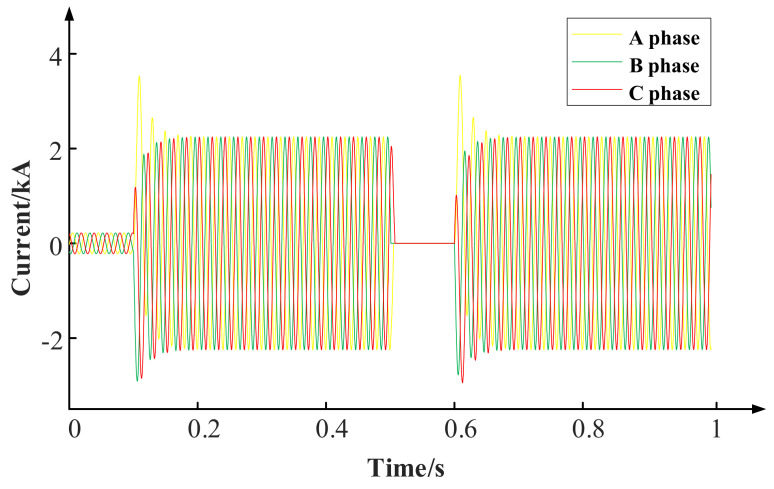
Burst short circuit and reclosing current waveform.

The time of reclosing is as follows: The transformer starts to operate normally, and the three-phase short-circuit fault occurs at 0.1 s, and it is a permanent short-circuit fault. The 0.5 s circuit breaker operates in tripping and cuts out the fault part. The 0.6 s circuit breaker operates in closing and the closing angle is 0°. The current waveform of the process is shown in Figure 3.

It can be seen from the simulation calculation that the secondary short-circuit impulse current is slightly larger than the primary short−circuit impulse current, which is due to the existence of the hysteresis effect in the opening process, so that there will be remanent magnetism inside the core and it is difficult to disappear in a short time. When the remanent magnetism and the direction of excitation are the same, the secondary short-circuit impulse current will be greater than the primary short-circuit impulse current. According to the current calculation model of the reclosing process established above, different closing angles are respectively set to calculate the impact current of a secondary short circuit under a three-phase short-circuit condition.

#### 2.1.2. Simulation of Short-Circuit Stress in Magnetic-Structure Coupling

The electromagnetic force received by the wire cake element of the finite element model of the transformer in the electromagnetic field is [14,15]:

(1) $$F_{i}=BSJ2\pi R$$

In the formula, F_i_ is the electromagnetic force received by the i unit of the winding, B is the magnetic induction intensity of the wire cake unit, S is the area of the wire cake unit, J is the current density of the wire cake unit, and R is the distance between the center line of the core and the center of gravity of the wire cake unit.

The total electromagnetic force of the cake is:

(2) $$F=\sum_{i=1}^{N}F_{i}$$

Through coupling, the electromagnetic force is added to the physical field of solid mechanics as a load. According to the elastic-plasticity of copper material, the electromagnetic force load is divided into several increments, and the balance equation of the finite element system based on increments is established in the physical field of solid mechanics:

(3) $$K_{ep}\Delta a=\Delta Q$$

where K_ep_ is the elastic-plastic stiffness matrix of the system, the incremental displacement phasor, and the unbalanced force phasor.

According to the constitutive relation of the material:

(4) Δε=BΔa

where: B is the strain matrix of the unit and is the strain increment.

(5) $$\Delta \sigma =D_{e}\Delta \varepsilon +\int D_{ep}d\varepsilon$$

where D_e_ is the elastic matrix, D_ep_ is the elastic–plastic matrix, and is the stress increment.

**Figure 4:**
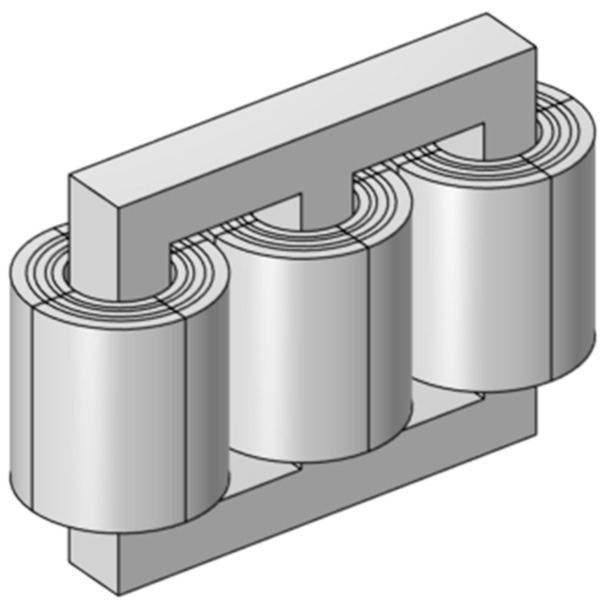
Transformer geometry model.

In order to calculate the short-circuit stress on the winding during reclosing, a three-dimensional magneto-circuit-force coupling finite element simulation model was established [16,17]. The following dimensions should be indicated in Figure 4. “Among them, the center distance between the columns is 1335 mm, the height of the core window is 1670 mm, the diameter of the core is 600 mm, the inner diameter of the low-pressure winding is 331 mm, the outer diameter of the low-pressure winding is 396 mm, the inner diameter of the medium-pressure winding is 435 mm, the outer diameter of the medium-pressure winding is 500 mm, the inner diameter of the high-pressure winding is 547 mm, and the outer diameter of the high-pressure winding is 640.5 mm.”

The impulse current calculated by the reclosing analog circuit is used as the excitation and input into the three-dimensional magneto-circuit-force coupling finite element simulation model for coupling calculation, and the short-circuit stress distribution of the winding is obtained.

**Figure 5:**
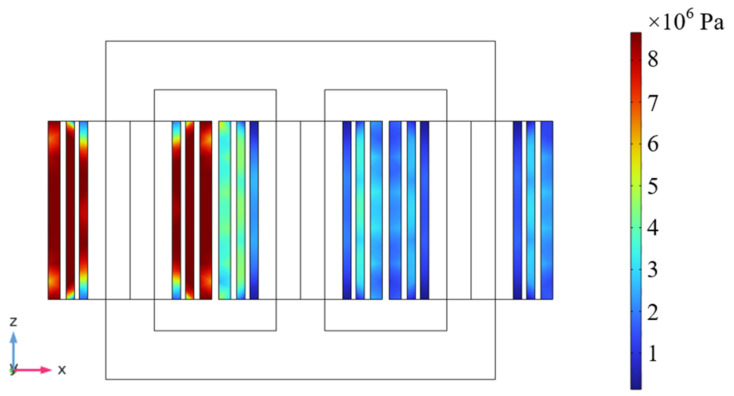
Short-circuit stress distribution diagram at 0° closing Angle.

Figure 5 shows the distribution of short-circuit stress when the closing angle is 0°, and Table 2 shows the short-circuit stress at different closing angles. When the closing angle is 0°, the maximum short-circuit stress of the insulating paper is 8.71 MPa. This test will take this as the basis for adding mechanical stress.

### 2.2. Oil–Paper Insulated Electric Field Simulation

Because the main insulation structure of the transformer can be approximated as a concentric cylinder with good symmetry, the two-dimensional model can meet the requirements when conducting an electric field simulation calculation. The two-dimensional simulation model established according to the actual parameters of the transformer is mainly composed of low-voltage winding, medium-voltage winding, high-voltage winding, insulating oil, insulated paper tube, and Angle ring, among which the low-voltage winding, medium-voltage winding and high-voltage winding are modeled in the unit of wire cake. The values of the relative dielectric constants of the insulation materials are shown in Table 3 [18].

**Figure 6:**
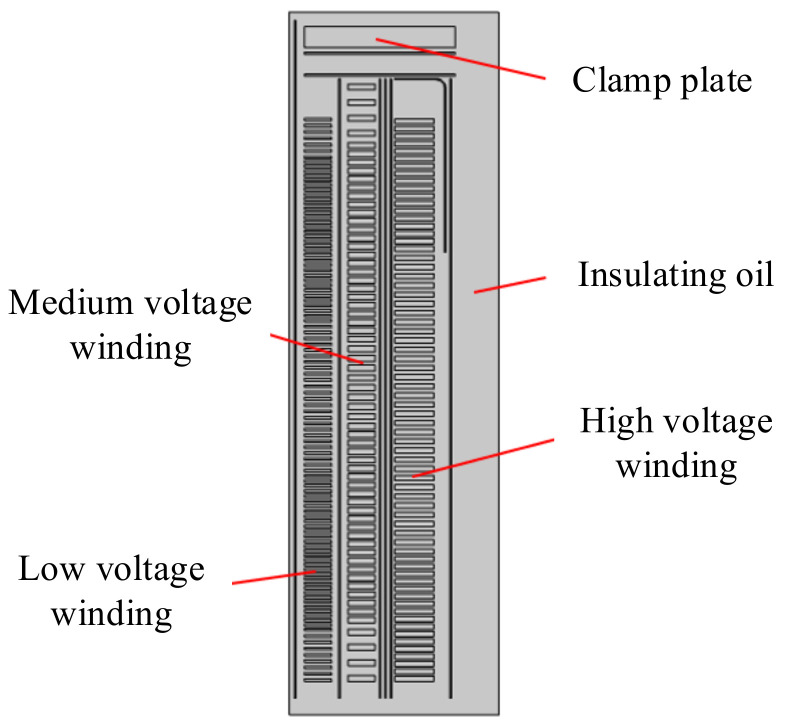
Calculation model of transformer main insulation.

**Figure 7:**
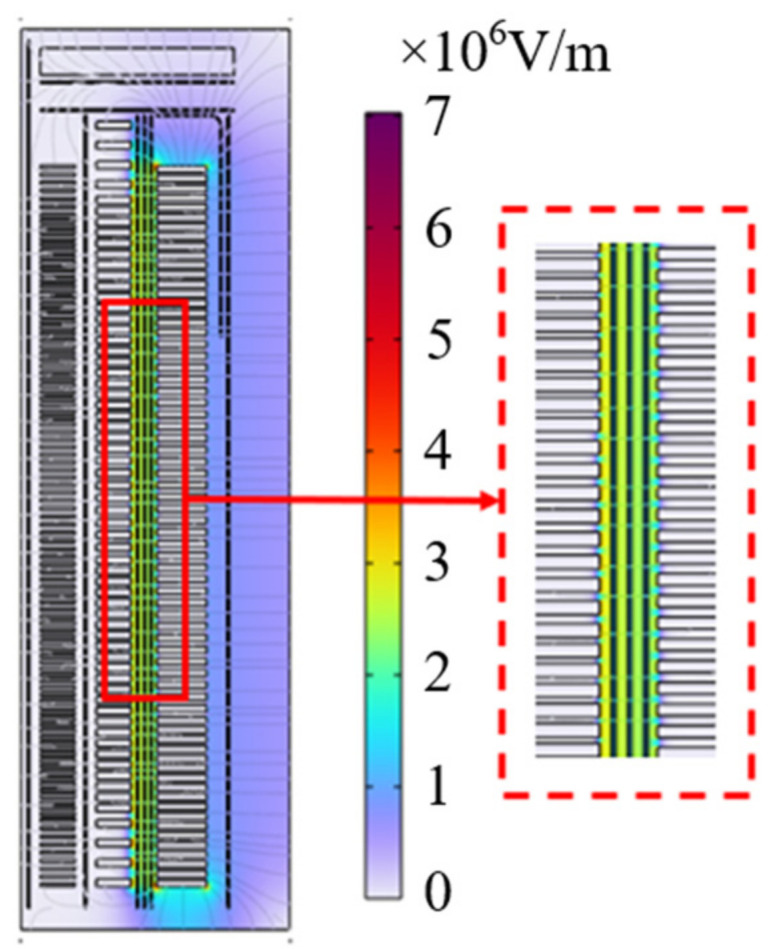
Distribution result of composite electric field of transformer main insulation.

The established calculation model of the main insulation of the transformer winding is shown in Figure 6. The ground potential is set at the boundary of the model (a homogeneous boundary condition), 110 kV voltage is applied in the high-voltage winding, and the ground potential is also set in the middle-voltage and low-voltage winding. The calculated distribution results of the transformer main insulation composite electric field are shown in Figure 7.

As can be seen from Figure 7, the distribution of the electric field in the middle of the transformer between the high- and middle-voltage windings is close to uniform, roughly between 2 and 3 × 10^6^ V/m. The average field strength between turns of the transformer is 2.26 × 10^6^ V/m through simulation calculation. This test is to explore the aging characteristics of insulating paper, so the average field strength between turns is taken as the basis for pressurization.

### 2.3. Oil–Paper Insulation Temperature Field Simulation

**Figure 8:**
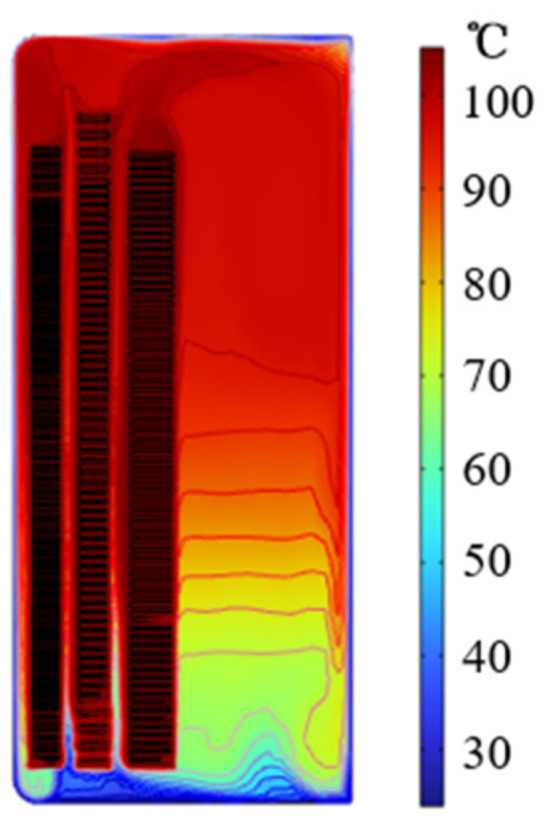
Transformer temperature distribution.

Taking a three-phase three-column true-type transformer as the research object, in order to simplify the calculation, the following assumptions are made when establishing the two-dimensional model of the oil-immersed transformer [19]:

- Do not consider the clamp, oil tank, magnetic shield, and other structural parts, mainly including transformer oil, iron core, and each winding;
- An axisymmetric model is adopted, and only the core and winding on one side of the axis are taken;
- The thermal conductivity, density, and specific heat capacity of the transformer core and winding are constant and do not change with the change in temperature;
- The ambient temperature is assumed to be 293 K. The two-dimensional calculation results of the transformer temperature field are shown in Figure 8.

As can be seen from Figure 8, the transformer winding temperature is high, the transformer oil temperature is stratified, and the temperature gradually decreases from top to bottom. The maximum average temperature of the winding during a short circuit is 107.67° C.

At the same time, according to GB/T 1094.5-2008 [20], the average thermal stability temperature of transformer winding under a short circuit is calculated theoretically. GB/T 1094.5-2008 specifies that power transformers should be free from damage under the action of short-circuit overcurrent caused externally. It is specified that the duration of the current to withstand the short-circuit heat resistance is 2 s, and the calculation formula of the average thermal stability temperature of the copper winding after the transformer burst short circuit is given as follows:

(6) $$\theta_{1}=\theta_{0}+2\times (235+\theta_{0})/(106000/(J^{2}t)-1)$$

where θ_1_ is the average temperature after winding short circuit t, unit °C; and θ_0_ is the starting temperature of the winding, which is the sum of the maximum ambient temperature and the average temperature rise limit of the winding, in unit °C. J is the short-circuit current density calculated according to the square mean root value of the symmetric short-circuit current, in A/mm^2^; and t is the short circuit duration, unit s.

Taking a three-phase three-column true-type transformer as the research object, the cross-sectional area of each winding wire of the transformer is given in Table 4.

When the high-voltage power supply is a short circuit, the short-circuit current value of the high voltage is 1542.3 A, and the short-circuit current value of the medium voltage is 4738.2 A. The high-voltage power supply, low-voltage short circuit, and high-voltage short-circuit current value is 880.9 A; the low-voltage short-circuit current value is 5727.4 A.

Taking high-voltage power supply and medium-voltage short-circuit working condition as an example, the short-circuit current calculation result is 1542.3 A, the cross-sectional area of the high-voltage winding is 61.44 mm^2^, then the symmetric short-circuit current density J of the transformer high-voltage winding is the ratio of 25.10 A/mm^2^, θ_0_ is 105 °C, t is 2 s, and the Formula (6) is replaced:

(7) $$\theta_{1}=105+\frac{2\times (235+105)}{\left(106000/(25.1^{2}\times 2)-1\right)}=113.2\;\mathrm{{}^{\circ}C}$$

Through the above calculation method, the average thermal stability temperature of the high- and low-voltage windings can be calculated, as shown in Table 5.

Through simulation, the maximum average temperature of winding under dynamic stability is 107.67 °C, and through theoretical calculation, the maximum average temperature of winding under thermal stability is 113.7 °C. Considering the maximum temperature condition that insulating paper may withstand [21], the maximum temperature of 113.7 °C is taken as the temperature condition of joint aging.

## 3. Electrical, Thermal, and Mechanical Aging Test of Oil–Paper Insulation

In order to study the performance aging law of insulating paper during transformer operation, the combined electrical, thermal, and mechanical aging test was carried out.

### 3.1. Electrical, Thermal, and Mechanical Aging Test Process of Oil–Paper Insulation

**Figure 9:**
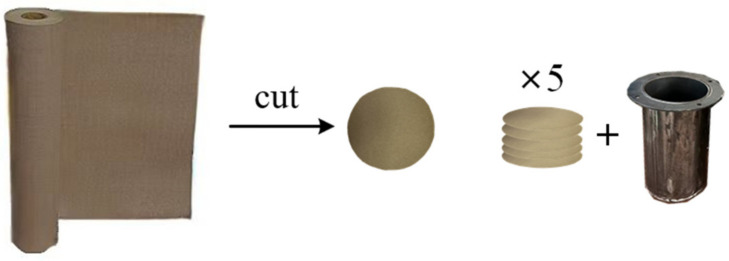
The process of cutting insulating paper and loading it into the oil tank.

**Figure 10:**
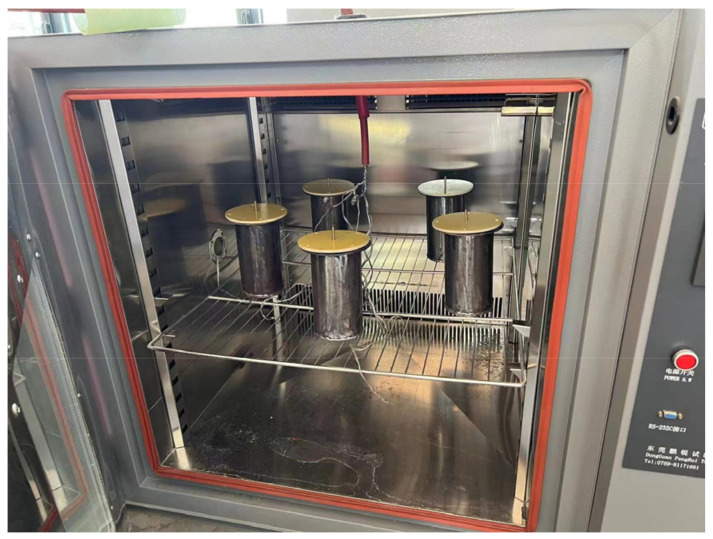
Test site drawing.

In order to reduce the moisture content in the insulating paper to a lower level, the insulating paper sample should be dried. First, the insulating paper (thickness 0.3 mm) should be cut into an electrode shape and placed in a beaker, and then the beaker should be placed in a vacuum drying oven at 105 ± 5 °C for 24 h. Taking into account the test error of a single-layer sample, five layers of insulating paper are put into the test oil tank, and 25# transformer oil is injected. This process is shown in Figure 9. Electrodes on the upper and lower ends of the insulating paper are installed (the electrodes are connected to the power frequency high-voltage test console), five groups of samples are set and numbered respectively in the oil tank, and finally, the oil tank is put into the high- and low-temperature-alternating humid heat test tank. The test site diagram is shown in Figure 10.

At the same time, 3.39 kV power frequency voltage is applied on both sides of the electrode to make the field strength between electrodes reach 2.26 × 10^6^ V/m. The temperature is set to 113.7 °C; the sampling time of the test was 0 h, 24 h, 48 h, 96 h, 144 h, and 192 h. After aging, 8.71 MPa equivalent stress was applied to the insulating paper to simulate the mechanical stress subjected to the insulating paper.

The test was divided into two groups, one group applied only electric field and temperature conditions, called the electro-thermal combined aging group, and the other group applied electric field, temperature, and mechanical stress conditions, and was called the electro-thermal combined aging group.

### 3.2. Aging Parameter Measurement of Oiled Paper Insulation

The mechanical and electrical properties of insulating paper will deteriorate to different degrees under the condition of multi-element aging. In this section, the degree of polymerization, tensile strength, and elongation at break are selected to reflect the change in mechanical properties during aging. Dielectric loss factor and breakdown voltage were selected to reflect the change in electrical performance during aging.

#### 3.2.1. Degree of Polymerization

The most intuitive way to reflect the aging of transformer oil–paper insulation is to detect the degree of polymerization of insulating paper. Insulating paper is the main reliance of oil-immersed transformer solid insulation. Cellulose composed of a glucose monomer connected to each other is the main component of insulating paper [22]. The number of glucose monomers is the Degree of Polymerization. The average degree of polymerization of insulating paper is generally measured by the viscosity method. According to GB/T 1548-2004 [23], the average degree of polymerization of insulating paper is calculated by measuring the viscosity of insulating paper [24].

Firstly, the insulating paper is degreased to eliminate the influence of transformer oil in the insulating paper on viscosity measurement. Then, the degreased insulating paper is broken into fibers in water, and copied into quantitative pulp sheets on the hand paper machine before air drying. Finally, the insulated pulp sheet was immersed in 25 mL distilled water for 0.5 h, and then 25 mL copper ethylenediamine solution with a concentration of 1 mol/L was added, stirring to obtain the concentrated solution with the viscosity to be measured, and the viscosity of the cellulose solution was measured by using an Ulster viscometer.

Relative viscosity of cellulose solution:

(8) $$\eta_{s}=\frac{T_{s}-T_{0}}{T_{0}}$$

where T_s_ is the cellulose solution flow time, and T_0_ is the solvent flow time.

Water content of the insulating paper:

(9) $$H=\frac{m-m_{0}}{m}$$

where m is the quality of the insulating paper before drying, and m_0_ is the quality of the insulating paper after drying.

Cellulose solution concentration:

(10) $$c=\left(m\frac{100}{45}\right)/\left(\frac{1}{1+H}\right)$$

According to the relative viscosity, the value of the characteristic viscosity is found, and the average degree of polymerization of the insulating paper is obtained according to formula (11).

(11) $$DP_{V}^{\alpha }=\frac{\left[\eta \right]}{K}=\frac{\left[\eta \right]\cdot c}{K\cdot c}$$

In the formula: K is 7.5 × 10^−3^.

#### 3.2.2. Tensile Strength and Elongation at Break

A mechanical universal testing machine was used to test the tensile strength of insulating paper according to GB/T 12914-2018 standard [25]. The sample size was 50 mm × 15 mm, and the drawing speed was 5 mm/min. The tensile strength and elongation at break were used as the evaluation basis.

Tensile strength refers to the maximum tension that the specimen can withstand before breaking per unit width under specified test conditions [26]. The calculation formula is:

(12) $$S=\frac{\bar{F}}{L_{W}}$$

where $\bar{F}$ is the average tensile strength and $L_{W}$ is the width of the insulating paper.

Elongation at break represents the ratio of the elongation of the specimen subjected to tension to fracture; that is, the ratio of the elongation length before and after stretching to the length before stretching. The formula is as follows:

(13) $$e=\frac{L_{a}-L_{0}}{L_{0}}$$

where $L_{a}$ is the length when pulled, $L_{0}$ is the original length.

#### 3.2.3. Dielectric Loss Factor

**Figure 11:**
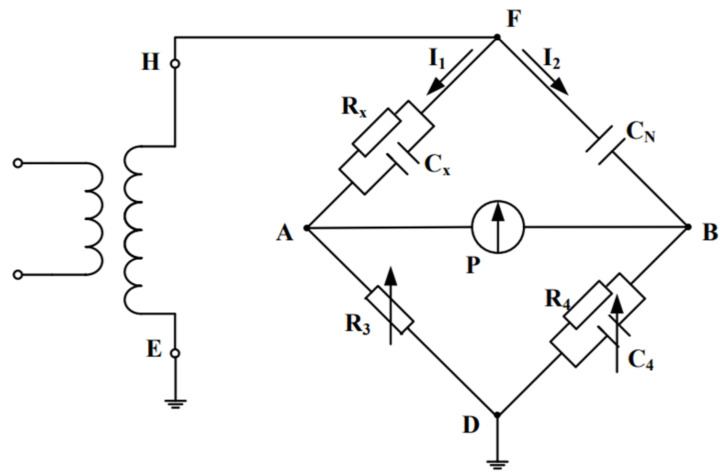
The Schering Bridge.

Dielectric loss factor tgδ is one of the basic indicators to characterize the insulation performance of objects. It is a characteristic parameter to characterize the specific loss of insulators under the action of alternating voltage, and has nothing to do with the shape and size of the insulators [27]. In this paper, a dielectric loss tester is used for measurement. The basic principle is the Schering bridge method. The diagram of the Schering bridge is shown in Figure 11.

In Figure 11, R_x_ and C_x_ represent the equivalent resistance and capacitance of the subject. By adjusting R_3_ and C_4_, the bridge reaches the balance, which is shown as the current of galvanometer P is zero. At this time:

(14) $$\frac{1}{\frac{1}{R_{X}}+j\omega C_{X}}\cdot \frac{1}{\frac{1}{R_{4}}+j\omega C_{4}}=R_{3}\cdot \frac{1}{j\omega C_{N}}$$

Equivalent resistance and capacitance of the subject can be obtained:

(15) $$C_{X}=\frac{R_{4}C_{N}}{R_{3}\left(1+\omega^{2}C_{4}^{2}R_{4}^{2}\right)}$$

(16) $$R_{X}=\frac{R_{3}\left(1+\omega^{2}C_{4}^{2}R_{4}^{2}\right)}{\omega^{2}C_{4}R_{4}^{2}C_{N}}$$

Dielectric loss factor of the subject:

(17) $$tg\delta =\frac{1}{\omega C_{X}R_{X}}=\omega C_{4}R_{4}$$

#### 3.2.4. Breakdown Voltage

When the electric field intensity applied at both ends of the dielectric exceeds a certain critical value, a small number of freely moving carriers inside the dielectric move violently, colliding with atoms on the lattice, ionizing, and destroying the molecular structure, resulting in the final breakdown; this phenomenon is called electrical breakdown [28].

**Figure 12:**
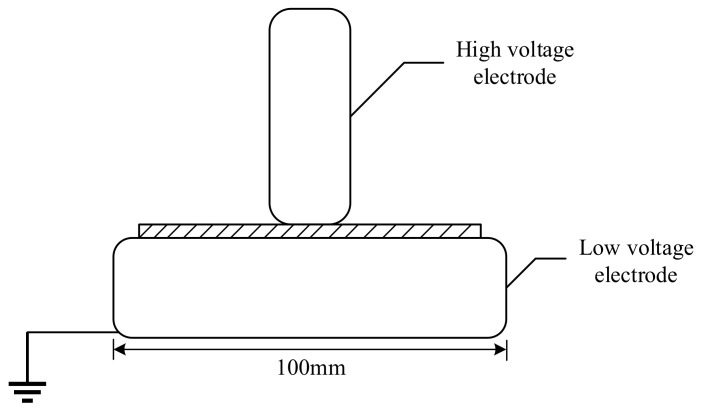
Unequal diameter electrode.

The 20 s step-up voltage method was used to perform the voltage withstand test on the single-layer insulating paper. As shown in Figure 12, the test electrodes were designed according to GB/T 1408.1-2016 [29], and electrodes with different diameters were used: Apply to the sample at a short-time breakdown voltage of 40%. If the sample withstands this voltage for 20 s without breakdown, the voltage shall be increased step by step. Each increase in voltage should be immediately and continuously increased for 20 s until breakdown occurs.

## 4. Aging Test Results and Analysis of Oil–Paper

### 4.1. Degree of Polymerization of Insulating Paper

**Figure 13:**
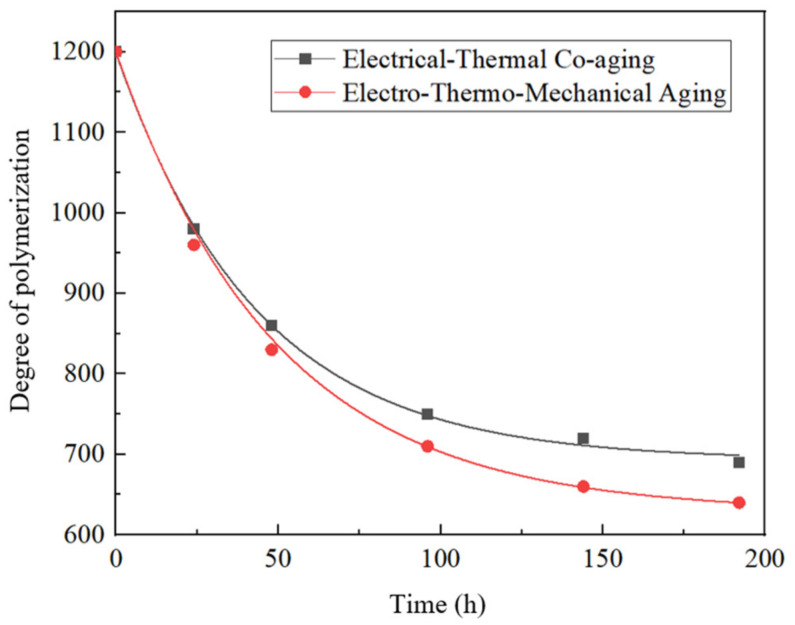
Insulating paper polymerization degree and aging time fit curve.

At different aging stages, the polymerization degree of the sample insulating paper in the electro-thermal combined aging group and the electro-thermal combined aging group is respectively measured as shown in Figure 13, and the change rate relative to the non-aging group is shown in Table 6.

Combined with Figure 13 and Table 6, it can be seen that with the extension of aging time, the degree of polymerization of insulating paper gradually decreases, and the decline rate decreases from fast to slow. This is related to the microstructure of cellulose. In the early stage of aging, the cellulose 1, 4-β glucoside bond in the amorphous region is first damaged and broken, and the amorphous region is easily damaged, so the degree of polymerization decreases rapidly. In the middle and late stages of aging, the 1, 4-β glucoside bond in the amorphous region has basically been consumed, which has little impact on the degree of polymerization. At this time, the degradation extends to the crystalline region, where the degradation of cellulose is more difficult, so the degree of polymerization decreases slowly. Compared with the two groups of samples, the decreased rate of polymerization degree in the electro-thermal and mechanical aging group was significantly higher than that in the electro-thermal aging group, indicating that the effect of mechanical stress accelerated the aging of insulating paper.

### 4.2. Tensile Strength and Elongation at Break of Insulating Paper

**Figure 14:**
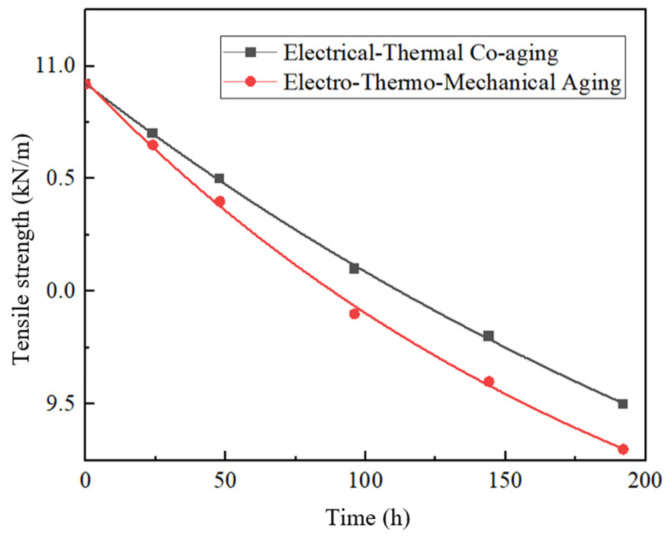
The fitting curve of tensile strength and aging time of insulating paper.

**Figure 15:**
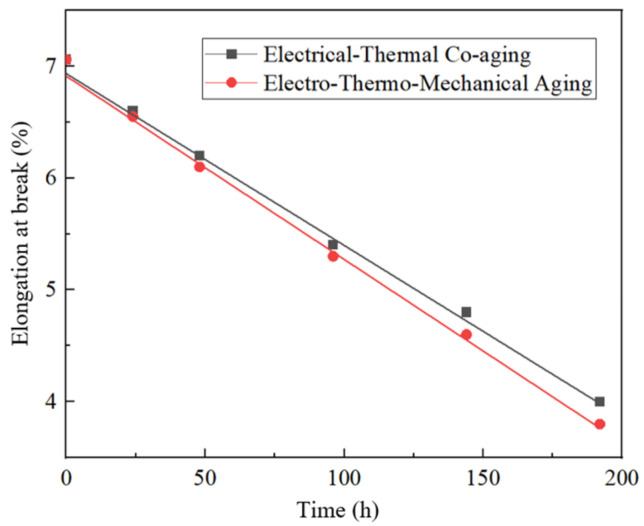
The elongation of insulating paper at breaking is fitted with the aging time curve.

The cellulose in the insulating paper will break with aging, reducing the mechanical strength. Therefore, the tensile strength and elongation at break of the aging insulating paper was tested, as shown in Figure 14 and Figure 15. Table 7 and Table 8 show the change rate of the aging insulating paper compared with that of the non-aging insulating paper.

Combined with Figure 14 and Figure 15 and Table 7 and Table 8, it can be seen that the tensile strength and elongation at break of the insulating paper of the electro-thermal group and the electro-thermal–force group decrease with the increase in aging time, and the tensile strength of the insulating paper of the electro-thermal–force group decreases faster [30]. It may be due to the destruction of molecular bonds between cellulose under the action of the electric field and high temperature, which leads to the decrease in tensile strength and elongation at break. After the application of mechanical force, the decreased rate of tensile strength and elongation at break is accelerated, which may be due to the effect of force, which further aggravates the destruction of molecular bonds between cellulose.

### 4.3. Dielectric Loss Factor of Insulating Paper

**Figure 16:**
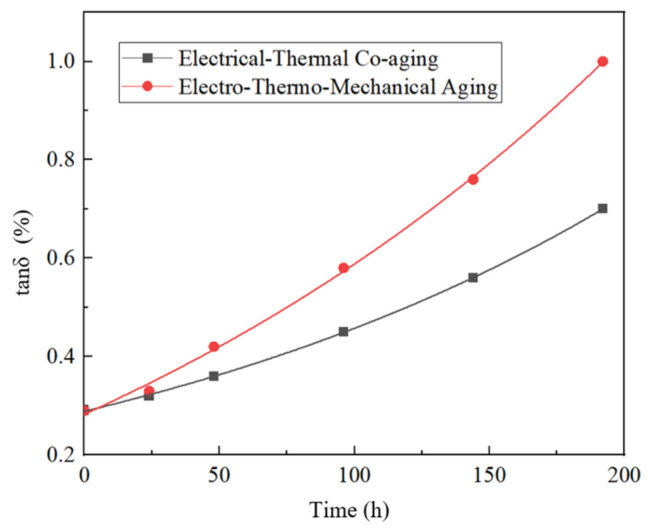
The fitting curve of dielectric loss factor and aging time of insulating paper.

A dielectric loss tester was used to measure the dielectric loss factors of the electro-thermal combined aging group and the electro-thermal combined force aging group, respectively. The results are shown in Figure 16, and the relative change rate of the dielectric loss in the non-aging group is shown in Table 9. 

In combination with Figure 16 and Table 9, it can be seen that the dielectric loss factor of insulating paper increases with the aging process, and the dielectric loss factor of the combined electric–heat–force aging group increases more significantly. This is because with the extension of the aging time of insulating paperboard aging deterioration, the dielectric loss increases, the electric–heat–force group compared to the electric–thermal group produces more mechanical stress, making the aging more rapid, and the dielectric loss factor increases faster.

### 4.4. Breakdown Voltage of Insulating Paper

**Figure 17:**
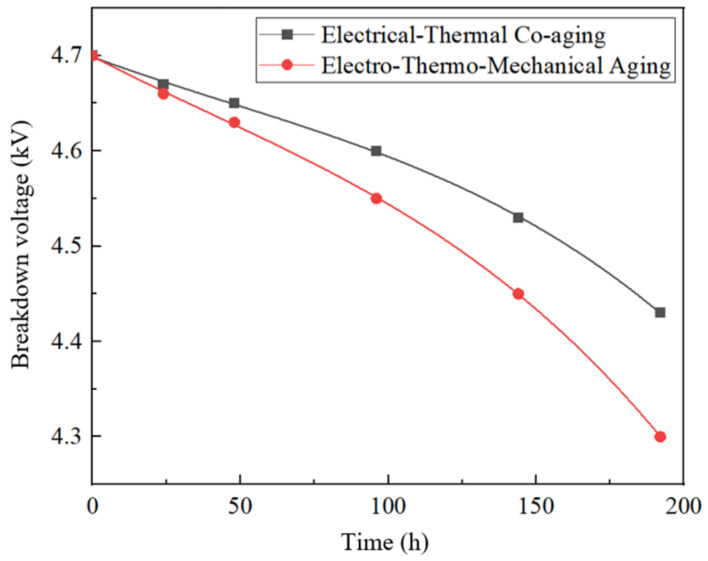
The breakdown voltage and aging time of insulating paper are fitted.

The insulating paper is dried at 90 °C for 2 h, the oil stains on the insulating paper are removed, and the pressure resistance test is carried out on the single-layer insulating paper (0.3 mm) with air as the medium. The breakdown voltage values of the single-layer insulating paper in the electro-thermal combined aging and electro-thermal combined mechanical aging groups were measured, respectively, and the results are shown in Figure 17. The relative change rate of the single-layer insulating paper in the non-aging group is shown in Table 10.

Combined with Figure 17 and Table 10, it can be seen that the breakdown voltage of insulating paper decreases with the increase in aging time, and the decline is slower in the early stage of aging and faster and faster in the later stage. This may be because under the action of the electric field and high temperature, the defects inside the insulating paper are expanded, and the breakdown process of the oil–paper insulation is to find the “weakest link” of the oil–paper insulation system. The breakdown voltage of the electric–thermal–force group drops faster than that of the electric–thermal group. This may be due to the fact that under the action of mechanical forces, the internal defects of the insulating paper are further expanded, and breakdown is more likely to occur.

## 5. Conclusions

In this paper, the electric field, temperature, and stress conditions of the insulating paper of a 110 kV transformer during the operation were calculated by modeling, and the corresponding aging test was carried out accordingly. Finally, the performance parameters of insulating paper in different aging stages were measured, and the following conclusions were drawn:

- In order to effectively and reasonably simulate the most severe real aging process of transformer insulating paper, a reclosing current calculation model, a magneto-circuit-force multiple physical field coupling model, and a simulation model of electrostatic field and temperature field are jointly established. The calculated field strength exerted by aging is 2.26 × 10^6^ V/m, the applied mechanical stress is 8.71 MPa, and the temperature is 113.7 °C.
- The increase in aging time, the degree of polymerization, tensile strength, and breakdown voltage all decreased, and the decrease was faster in the electro-thermal–mechanical aging group than in the electro-thermal aging group. The dielectric loss factor increases, and the increase in the electro-thermal and mechanical aging group is faster than that of the electro-thermal aging group. This shows that the mechanical force has an accelerated effect on the failure of the mechanical and electrical properties of the insulating paper.
- Within 24 h, the difference between the performance change rates of the electro-thermal combined aging group and the electro-thermal combined aging group is less than 5%, but with the extension of aging time, the performance deterioration degree introduced by force aging is higher, which indicates that the mechanical damage resistance, that is, the short-circuit resistance, of the insulating paper is significantly reduced after a long time of aging.

## Figures and Tables

**Table 1:** Main parameters of transformer.

Parameter Name	Numerical Value	Parameter Name	Numerical Value
P_k(1-2)_/kW	147	U_k(1-2)_/%	10.03
P_k(3-1)_/kW	143.5	U_k(3-1)_/%	17.14
P_k(2-3)_/kW	120	U_k(2-3)_/%	6.67
P_0_/kW	40.24	I_0_/%	0.9

**Table 2:** Short-circuit stress at different closing angles.

Closing Angle/°	0	45	90	135	180
**Short-circuit stress/MPa**	8.71	6.84	5.68	6.86	8.53

**Table 3:** The relative dielectric constant of the insulating material.

The Insulation Material Involved in the Model	Relative Dielectric Constant
Insulating paper (turn insulation, Angle ring)	3.2
Insulated paper tube	4.4
Insulating oil	2.2

**Table 4:** Cross-sectional area of each winding conductor of the transformer.

Winding Type	Traverse Area/mm^2^
High-voltage winding	61.44
Medium-voltage winding	182.75
Low-voltage winding	291.27

**Table 5:** Average thermal stability temperature of each transformer winding.

Winding Type	Thermally Stable Mean Temperature/°C
High-voltage winding	113.2
Medium-voltage winding	113.7
Low-voltage winding	110

**Table 6:** Changing rate of polymerization degree of insulating paper.

Time/h	Electro-Thermal	Electric–Heat–Force
0	0	0
24	−18.3%	−19.9%
48	−28.3%	−30.9%
96	−37.1%	−40.7%
144	−39.6%	−44.8%
192	−42.9%	−46.7%

**Table 7:** Changing rate of tensile strength of insulating paper.

Time/h	Electro-Thermal	Electric–Heat–Force
0	0	0
24	−2.01%	−2.47%
48	−3.85%	−4.76%
96	−7.51%	−9.34%
144	−10.3%	−12.1%
192	−12.9%	−14.8%

**Table 8:** Change rate of elongation of insulating paper when broken.

Time/h	Electro-Thermal	Electric–Heat–Force
0	0	0
24	−6.52%	−7.22%
48	−12.2%	−13.6%
96	−23.5%	−24.9%
144	−32.0%	−34.8%
192	−43.3%	−46.2%

**Table 9:** Dielectric loss factor change rate of insulating paper.

Time/h	Electro-Thermal	Electric–Heat–Force
0	0	0
24	10.3%	13.8%
48	24.1%	44.8%
96	55.2%	100%
144	93.1%	162.1%
192	141.4%	244.8%

**Table 10:** Breakdown voltage change rate of insulating paper.

Time/h	Electro-Thermal	Electric–Heat–Force
0	0	0
24	−0.64%	−0.85%
48	−1.06%	−1.49%
96	−2.13%	−3.19%
144	−3.62%	−5.32%
192	−5.74%	−8.51%

## Data Availability

The experimental data will not be disclosed.
